# Performance of Digital Contact Tracing Tools for COVID-19 Response in Singapore: Cross-Sectional Study

**DOI:** 10.2196/23148

**Published:** 2020-10-29

**Authors:** Zhilian Huang, Huiling Guo, Yee-Mun Lee, Eu Chin Ho, Hou Ang, Angela Chow

**Affiliations:** 1 Department of Clinical Epidemiology Office of Clinical Epidemiology, Analytics, and Knowledge Tan Tock Seng Hospital Singapore Singapore; 2 Department of Urology Tan Tock Seng Hospital Singapore Singapore; 3 Lee Kong Chian School of Medicine Nanyang Technological University Singapore Singapore; 4 Department of Ear, Nose, and Throat Tan Tock Seng Hospital Singapore Singapore; 5 Department of Emergency Medicine Tan Tock Seng Hospital Singapore Singapore

**Keywords:** infectious disease, real-time locating systems, electronic medical records, COVID-19, contact tracing, public health

## Abstract

**Background:**

Effective contact tracing is labor intensive and time sensitive during the COVID-19 pandemic, but also essential in the absence of effective treatment and vaccines. Singapore launched the first Bluetooth-based contact tracing app—TraceTogether—in March 2020 to augment Singapore’s contact tracing capabilities.

**Objective:**

This study aims to compare the performance of the contact tracing app—TraceTogether—with that of a wearable tag-based real-time locating system (RTLS) and to validate them against the electronic medical records at the National Centre for Infectious Diseases (NCID), the national referral center for COVID-19 screening.

**Methods:**

All patients and physicians in the NCID screening center were issued RTLS tags (CADI Scientific) for contact tracing. In total, 18 physicians were deployed to the NCID screening center from May 10 to May 20, 2020. The physicians activated the TraceTogether app (version 1.6; GovTech) on their smartphones during shifts and urged their patients to use the app. We compared patient contacts identified by TraceTogether and those identified by RTLS tags within the NCID vicinity during physicians’ 10-day posting. We also validated both digital contact tracing tools by verifying the physician-patient contacts with the electronic medical records of 156 patients who attended the NCID screening center over a 24-hour time frame within the study period.

**Results:**

RTLS tags had a high sensitivity of 95.3% for detecting patient contacts identified either by the system or TraceTogether while TraceTogether had an overall sensitivity of 6.5% and performed significantly better on Android phones than iPhones (Android: 9.7%, iPhone: 2.7%; *P*<.001). When validated against the electronic medical records, RTLS tags had a sensitivity of 96.9% and specificity of 83.1%, while TraceTogether only detected 2 patient contacts with physicians who did not attend to them.

**Conclusions:**

TraceTogether had a much lower sensitivity than RTLS tags for identifying patient contacts in a clinical setting. Although the tag-based RTLS performed well for contact tracing in a clinical setting, its implementation in the community would be more challenging than TraceTogether. Given the uncertainty of the adoption and capabilities of contact tracing apps, policy makers should be cautioned against overreliance on such apps for contact tracing. Nonetheless, leveraging technology to augment conventional manual contact tracing is a necessary move for returning some normalcy to life during the long haul of the COVID-19 pandemic.

## Introduction

The fight against COVID-19 is expected to be a long haul due to its high transmissibility, despite its low virulence rates [1]. There have been predictions of potential second waves of SARS-CoV-2 infections, and several countries have experienced repeated waves of outbreaks after seemingly successful efforts of containing COVID-19 transmissions [2,3]. In the absence of effective treatment or vaccination, approaches to preventing transmissions have included social distancing, use of face masks, hygiene measures (eg, disinfection), case isolation, contact tracing, and quarantine [1]. Effective contact tracing is essential for preventing transmissions and subsequent generations of SARS-CoV-2 infections [4].

Contact tracing is the process of identifying and subsequently acquiring information from persons potentially exposed to infectious diseases [4]. Conventional contact tracing methods include a mixture of patient interviews and contact verifications to map the social and work encounters of an infected individual. These methods enable the contact tracer to obtain a comprehensive list of persons potentially exposed to infectious individuals [5,6]. Additional measures, such as patient and visitor registration system downloads, staff roster and electronic medical record (EMR) reviews, and direct observations via closed-circuit television have been employed for contact tracing in health care settings [7]. These processes are labor intensive, yet time sensitive and crucial for stemming the spread of the infectious disease at the emergence of an outbreak [4,8,9].

During the COVID-19 outbreak, contact tracing can be challenging in large communities with a limited pool of contact tracers [6,10]. Technology can potentially overcome this barrier by simplifying the process of data collection. Several health care institutions have benefitted from the use of a real-time locating system (RTLS) for contact tracing. Hellmich et al [11] were able to identify twice as many potential contacts of confirmed pertussis cases in an emergency department with RTLS compared with EMR review. Ho et al [12] also found that the use of location-based RTLSs to complement EMR review increased the sensitivity of detecting health care workers’ contact with COVID-19 inpatients from 47.2% to 77.8%. However, despite the positive outcomes of using RTLSs for contact tracing [11-13], such technologies have largely been confined to health care institutions due to the high costs of infrastructure setup [14,15].

Contact tracing apps can provide a more feasible solution to the barrier of scale presented by large communities with high smartphone penetration rates. The ubiquity and relatively low developmental cost of smartphone apps allow for large-scale deployment in time-sensitive situations. Therefore, many countries have jumped on the bandwagon of contact tracing apps [16-19] to augment contact tracing capabilities in response to the COVID-19 pandemic. Likewise, Singapore launched its first Bluetooth-based contact tracing app—TraceTogether—in March 2020 [16,18]. The app is available on both the Google and Apple app stores, and approximately one-sixth of Singapore residents downloaded the app during the peak of the COVID-19 outbreak in April 2020 [20].

Despite the potential of contact tracing apps in enhancing contact tracing efforts during the COVID-19 pandemic, this potential can only be realized when 60% of the population use the app [6]. The efficacy of large-scale technology adoption depends on the law and enforcement measures of the country, public trust in personal data protection, and users’ perceived utility of adopting the technology, along with other factors [21-23]. Prior to large-scale technology adoption, the validity of novel contact tracing methods should be assessed to optimize the time and resources used in tackling the COVID-19 pandemic. The effectiveness of digital contact tracing tools has yet to be established due to the paucity of published data in real-life outbreak settings [24]. Hence, we compared the performance of the TraceTogether app with that of a wearable tag-based RTLS and validated both against the EMRs at the National Centre for Infectious Diseases (NCID), the national referral center that managed the majority of Singapore’s hospitalized COVID-19–positive patients in May 2020.

## Methods

### Setting

This study was conducted in the COVID-19 screening center of the NCID in Singapore. The NCID was the designated hospital for managing suspected and confirmed COVID-19 cases during the COVID-19 outbreak in Singapore. Physicians from the co-located Tan Tock Seng Hospital were deployed to the NCID screening center on 10-day rotating shifts to manage the center during the outbreak. Singapore entered a 2-month partial lockdown phase from April 7 through June 1, 2020 due to a surge in COVID-19 cases in the community and foreign worker dormitories.

The study was conducted over a 10-day physician-posting period from May 10 through May 20, 2020. At the time of the study, the NCID was managing more than 70% of Singapore’s COVID-19–positive cases. The majority of patients who attended at the screening center during the study period were residents of foreign worker dormitories.

### Study Design

**Figure 1:**
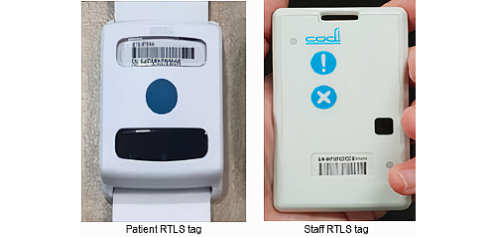
Mandatory real-time locating system (RTLS) tags worn by patients and staff within the vicinity of the COVID-19 screening centre for contact tracing purposes.

**Figure 2:**
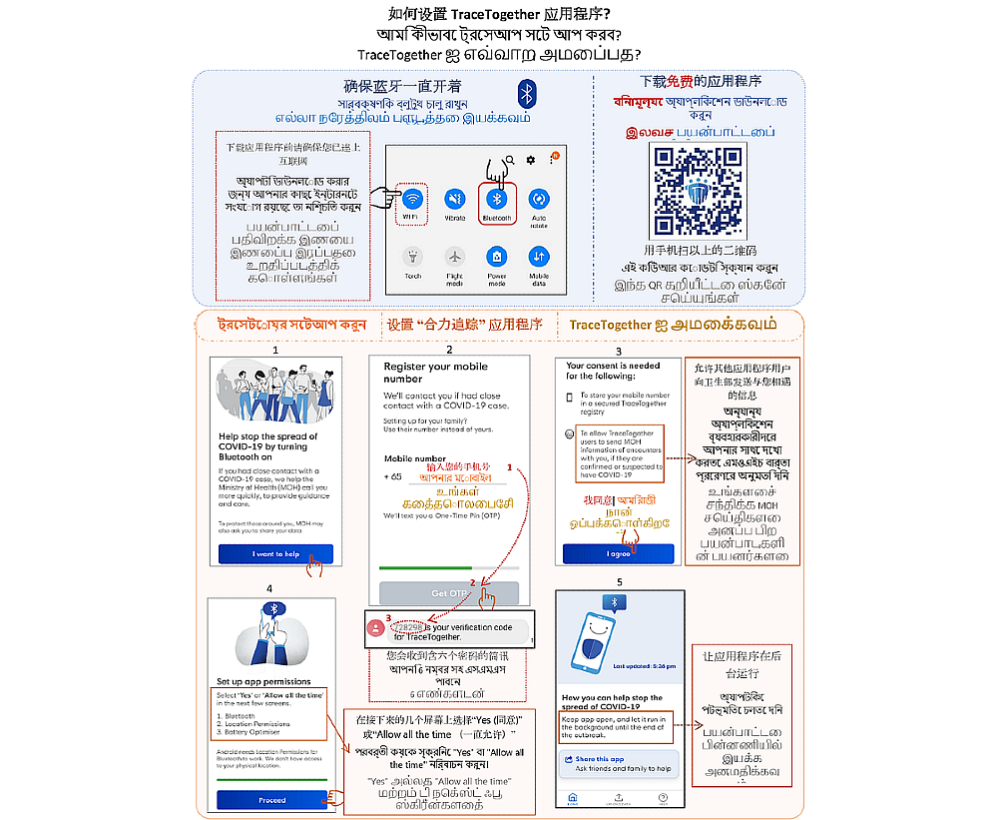
Pictorial instructions to download and activate version 1.6 of the TraceTogether app provided at the National Centre for Infectious Diseases COVID-19 screening centre, Singapore from May 10 through May 20, 2020.

We employed a cross-sectional study design to validate the TraceTogether app and the NCID’s wearable RTLS wrist tag against EMRs. All patients and physicians in the NCID screening center were issued temporary RTLS tags for contact tracing (Figure 1). Wearing the RTLS tags was mandatory for entry into the NCID screening center (Figure 1). Physicians deployed to the NCID screening center from May 10 to May 19, 2020 were instructed to install the TraceTogether app (version 1.6), activate their smartphone’s Bluetooth function during their shifts, and urge their patients to download and activate the TraceTogether app when they medically attended to their patients. Pictorial instructions to download and activate the TraceTogether app were available in English, Tamil, Bengali, and Mandarin at every screening station (Figure 2).

Each patient was assigned to a designated screening station (arranged 2 meters apart from other stations), at which the patient was medically attended. Patients could download the app while awaiting attendance by their physicians using the complimentary Wi-Fi at the screening center. Attending physicians also encouraged patients to download and activate the TraceTogether app.

### Tag-Based RTLS

The RTLS was fitted into the NCID building since the building’s official opening in September 2019. RTLS location exciters and wireless access points were fitted throughout the NCID building to detect signals from RTLS tags (CADI Scientific). The tag receives a signal whenever it passes a location exciter and sends a radio-frequency signal to the access points to determine the exact location of the RTLS tag. Concurrently, RTLS tags are enabled with tag-tag radio-frequency identification technology to determine close contacts of <=2 meters between staff and patients. Although the location-based radio-frequency identification system in the NCID has been validated by Ho et al [12], the performance of tag-based technology has yet to be assessed.

### TraceTogether App

The TraceTogether app was developed by a government-linked company—GovTech—in response to the COVID-19 outbreak. The free-to-download app worked by exchanging Bluetooth signals with other nearby app users and storing the encrypted data locally in the smartphone. Users were requested to upload the data captured on their smartphones should they be confirmed with COVID-19 infection to facilitate contact tracing. Data stored on the phone were automatically deleted after three weeks [17]. Version 1.6 of the app only stored the user’s mobile phone number as identity data and collected the phone number, proximity, and duration of users’ contacts. Both the mobile phone number and anonymized random user ID (generated from account creation) were stored in the secure GovTech server.

### EMR System

All staff providing clinical care to patients at the NCID were issued with rights to make entries in patients’ EMRs. Therefore, every patient screened at the NCID screening center had their clinical encounters recorded by the NCID EMR system. Hence, the EMR system was considered the gold standard for validating patient-physician contacts in this study.

### Participants

Data validation was only possible when TraceTogether and RTLS data from physicians were available. Therefore, only physicians who uploaded their TraceTogether data and had working RTLS tags were included as participants in this study. By the end of the 10-day posting period, 18 physicians with working RTLS tags had uploaded the data captured from the TraceTogether app from their smartphones to the cloud system.

### Data Analyses

We compared patient contacts identified by TraceTogether with those detected by the tag-based RTLS within the NCID screening center from 8:30 AM on May 10, 2020 through 8:30 AM on May 20, 2020 (Table 1). The mobile phone numbers of patients identified by the 18 participating physicians’ RTLS tags were compared with the mobile phone numbers captured by the physicians’ TraceTogether app. These mobile phone numbers were identified as those from the NCID patient registration (ie, from patients who attended the NCID during the study period).

We also reviewed the EMRs of 156 patients who attended the NCID screening center from 8:30 AM on May 12, 2020 to 8:30 AM on May 13, 2020 to determine if the eight participating physicians who were on shift during that time were among the attending physician(s) of each patient. Only records that fell within each physician’s shift were considered. The mobile phone numbers of these 156 patients were compared with the mobile phone numbers of patients identified by the eight physicians’ RTLS tags and TraceTogether app, respectively, to determine whether the respective technologies had detected them. We then computed the sensitivity, specificity, positive and negative predictive values, and likelihood ratios of the two digital technologies, using EMR review as the gold standard (Table 2).

**Table 1:** Comparison of the number of patient contacts identified by a tag-based real-time locating system within the vicinity of the NCID and the TraceTogether app on iPhones and Android phones.

Phone type and physician number		RTLS^a^	TraceTogether^b^	RTLS+TraceTogether	RTLS or TraceTogether	Sensitivity of RTLS^c^ (%)	Sensitivity of TraceTogether^d^ (%)
**iPhones**							
	1	65	0	0	65	100	0
	2	52	0	0	52	100	0
	3	2	0	0	2	100	0
	4	70	0	0	70	100	0
	5	59	4	1	64	93.8	7.8
	6	9	3	0	12	75	25
	7	14	0	0	14	100	0
	8	18	2	0	20	90	10
	9	59	0	0	59	100	0
	10	14	0	0	14	100	0
	Total	362	9	1	372	97.6	2.7^e^
**Android phones**							
	11	83	1	2	86	98.8	3.5
	12	61	0	0	61	100	0
	13	11	0	1	12	100	8.3
	14	78	5	2	85	94.1	8.2
	15	12	0	0	12	100	0
	16	80	12	6	98	87.8	18.4
	17	43	2	0	45	95.6	4.4
	18	42	10	3	55	81.8	23.6
	Total	410	30	14	454	93.4	9.7^e^
**iPhone+Android Phone**							
	Total	772	39	15	826	95.3	6.5

^a^RTLS: real-time locating system.

^b^TraceTogether-only data includes contacts that were concurrently detected by the TraceTogether app and the NCID location-based RTLS to ensure that the encounters were within NCID’s vicinity.

^c^RTLS sensitivity = (number of contacts detected by RTLS + (RTLS+TraceTogether))/(number of contacts detected RTLS or TraceTogether).

^d^TraceTogether sensitivity = (number of contacts detected by TraceTogether + (RTLS+TraceTogether))/(number of contacts detected by RTLS or TraceTogether).

^e^*P*<.001 when the sensitivity of TraceTogether was compared between Android phones and iPhones.

**Table 2:** Performance of the tag-based real-time locating system and TraceTogether app validated against electronic medical records.^a^

Digital contact tracing tool	Sensitivity^b^	Specificity^c^	Positive predictive value	Negative predictive value	LR+^d^	LR−^e^
Tag-based real-time locating system	96.9% (31/32)	83.1% (103/124)	59.6% (31/52)	99.0% (103/104)	5.73	0.04
TraceTogether app	0.0% (0/32)	98.4% (122/124)	0.0% (0/2)	79.2% (122/154)	0.00	1.02
Either digital contact tracing tool	96.9% (31/32)	81.5% (101/124)	57.4% (31/54)	99.0% (101/102)	5.24	0.04

^a^Physicians 1, 4, 5, 8, 9, 11, 12, and 18 from Table 1 were included in the Table 2 analyses.

^b^Denominator for sensitivity = number of patients attended by eight physicians who uploaded their TraceTogether data and had a work shift between 8:30 AM on May 12, 2020 and 8:30 AM on May 13, 2020.

^c^Denominator for specificity = number of patients attended by other physicians in the same work shift between 8:30 on May 12, 2020 and 8:30 AM on May 13, 2020.

^d^LR+: Positive Likelihood Ratio; LR+ = sensitivity/(1 − specificity).

^e^LR−: Negative Likelihood Ratio; LR− = (1 − sensitivity)/specificity.

### Ethical Considerations

This study was approved by the National Healthcare Group Domain Specific Review Board in Singapore.

## Results

Table 1 shows the comparative performance of the RTLS and TraceTogether app in identifying patient contacts of individual physicians. The RTLS had a high sensitivity of 95.3% in detecting all patient contacts identified either by the RTLS system or TraceTogether app, while TraceTogether had an overall sensitivity of 6.5%. Version 1.6 of the app performed significantly better on Android phones than iPhones (Android: 9.7%, iPhone: 2.7%, *P*<.001).

Table 2 shows the performance of the tag-based RTLS and TraceTogether app validated against EMRs. RTLS tags had high sensitivity (96.9%) and specificity (83.1%), as the tags could detect patient contacts other than those between patients and the participating physicians who medically attended to them. TraceTogether detected only 2 patient contacts with physicians who did not attend to them. Hence, the app had a sensitivity of 0% and specificity of 98.4% in a clinical setting. The sensitivity of identifying patient contacts increased to 96.9% when both digital contact tracing tools were used.

The positive predictive value and negative predictive value of the RTLS were 59.6% and 99.0%, respectively, while those of TraceTogether were 0% and 79.2%, respectively. Positive and negative predictive values are influenced by the prevalence of the disease, which in this case, was the proportion of patients in contact with physicians. A higher prevalence likely leads to higher positive predictive values. The RTLS’s moderately high positive likelihood ratio of 5.73 and high negative likelihood ratio of 0.04 suggest that the RTLS is capable of ruling in and ruling out patient contacts.

## Discussion

Effective and timely contact tracing is essential in slowing the spread of COVID-19 in the community. We compared the performance of a contact tracing app—TraceTogether—and NCID RTLS tags in identifying patient-physician contacts and validated both digital contact tracing tools against the EMRs at the NCID COVID-19 screening center. To our knowledge, this is the first study to assess the validity of a contact tracing app in a real-life outbreak setting (ie, the COVID-19 pandemic).

TraceTogether had a much lower sensitivity than the tag-based RTLS in identifying patient contacts in a clinical setting. High sensitivity is preferred for digital contact tracing tools to rule out the possibility of failing to detect close contacts with COVID-19 cases. The low sensitivity of TraceTogether could be attributed to the small proportion of patients who activated the app and turned on their Bluetooth, despite physicians’ prompts and the reminder posters at the screening center. In contrast, all patients had RTLS tags attached to their wrists at registration. Hence, we were able to more accurately assess the validity of the RTLS tags in this study.

Although the tag-based RTLS performed well for contact tracing in a clinical setting, the high setup cost would render it less feasible for a community-wide scale-up. Distributing RTLS tags and enforcing their use in the community would be much more challenging than doing so in a clinical setting. Previous RTLS studies have been mainly confined to a defined setting [11,13,25,26]. On the other hand, Bluetooth technology is low cost, available on personal digital devices, and interoperable among Bluetooth-enabled devices [27]. This flexibility is essential for facilitating widespread adoption of contact tracing tools, as users would have the convenience of selecting their preferred form of the contact tracing tool.

A critical mass of app adoption must be achieved to increase the sensitivity and positive predictive value of TraceTogether [28]. Most people have probably not heard of the app or found it useful to download the app during the study period, as movement was restricted due to lockdown measures. The concept of contact tracing apps only actualized on a large scale after it was realized that the fight against COVID-19 was going to be a long haul. Privacy concerns regarding data storage and location tracking were likely the biggest barrier against such contact tracing apps [20,23,28]. Despite the hype surrounding contact tracing apps, health systems worldwide have not revolutionized contact tracing efforts for COVID-19. Partial regulatory enforcements, effective communication of app utility, and a good understanding of the barriers and facilitators of contact tracing app adoption are crucial for app adoption to reach a critical mass.

Given the uncertainty of the adoption and capabilities of contact tracing apps [22,29], contact tracers and policy makers should be cautioned against the overreliance on such apps for contact tracing. An infectious disease workgroup estimated that manual contact tracing would reduce COVID-19 transmissions by 61% compared with the 44% reduction provided by app-based tracing if 53% of the population uses the app [30]. Even if a contact tracing app achieved widespread adoption and high sensitivity in detecting contacts, manual verifications would still be required to ascertain the contact before actions can be undertaken to quarantine potential exposures. In reality, a mixture of contact tracing methods is required to optimize the performance of contact tracing [23].

There were limitations to this study. First, we were unable to enforce the usage of TraceTogether among all physicians at the screening center for a comprehensive review on its performance. We could only consider the patient contacts of physicians who uploaded their TraceTogether data to the cloud system. This limitation in a clinical setting reflects the challenges of wide-spread adoption of app-based contact tracing tools in the community. However, despite the difficulties in enforcing wide-spread adoption, app-based contact tracing can complement conventional contact tracing by speeding up the process. Second, the lackluster performance of TraceTogether version 1.6 on the iPhone may have decreased the performance of the app. Many patient contacts could have been missed, since half of the physicians in this study used an iPhone and the majority of the dormitory workers used an Android phone.

The performance of TraceTogether is expected to improve with app upgrades and increased use over time. We have provided feedback to the developers of TraceTogether, and the app has been upgraded to improve its performance on the iPhone. Since TraceTogether is Singapore’s national contact tracing app, many firms have encouraged its use [11] among employees who had to return to work after lockdown. App use has been made mandatory among dormitory workers [31] who have the highest risk of COVID-19 transmissions, and Bluetooth tokens have been distributed to populations susceptible to SARS-CoV-2 infection, such as older adults [32]. Factors that influence people’s willingness to adopt TraceTogether are being assessed to achieve higher adoption rates so that the digital contact tracing tool can be effective in the community.

In conclusion, technological and app adoption barriers must be overcome for digital contact tracing tools to be effective for contact tracing during the COVID-19 pandemic. Although the RTLS performed well for contact tracing in a clinical setting, its implementation will be confined to a defined setting. The sensitivity of contact tracing apps needs to be improved for app-based contact tracing to be viable in the community. Leveraging technology to complement conventional manual contact tracing is a necessary move for returning some normalcy to life after exiting lockdowns. The capabilities and utility of digital contact tracing tools are expected to grow over the long haul of the COVID-19 pandemic as the technology matures.

## Abbreviations

EMR: electronic medical record
NCID: National Centre for Infectious Diseases
RTLS: real-time locating system
